# Internalization of Mastery Goals: The Differential Effect of Teachers’ Autonomy Support and Control

**DOI:** 10.3389/fpsyg.2020.599303

**Published:** 2021-02-05

**Authors:** Moti Benita, Lennia Matos

**Affiliations:** ^1^Department of Education, Ben-Gurion University of the Negev, Beersheva, Israel; ^2^Department of Psychology, Ponifical Catholic University of Peru, Lima, Peru

**Keywords:** mastery goals, goal-complex, autonomy supportive teaching, controlling teaching, engagement, behavioral engagement

## Abstract

Two linked studies explored whether students’ perceptions differentiate between teachers’ autonomy support and control when presenting mastery goals, and the outcomes of these two practices, in terms of students’ internalization of mastery goals and their behavioral engagement. In two phases, Study 1 (*N* = 317) sought to validate a new instrument assessing students’ perceptions of teachers’ autonomy support and control when presenting mastery goals. Study 2 (*N* = 1,331) demonstrated that at both within- and between-classroom levels, perceptions of teachers’ autonomy support for mastery goals were related to students’ mastery goals’ endorsement and behavioral engagement. These relations were mediated by students’ autonomous reasons to pursue learning activities. Perceptions of teachers’ control predicted disengagement through controlled reasons for learning, but only at the within-classroom level. This research joins a growing body of work demonstrating that combining achievement goal theory with SDT can further our understanding of the underpinnings of achievement motivation. It suggests that if teachers want their students to endorse mastery goals (and be more engaged), they need to use more autonomy supportive practices and less controlling ones.

## Introduction

Achievement goal researchers (e.g., Dweck, 1986; Ames, 1992) have long maintained that teachers should promote mastery goals in the classroom, as these goals, once endorsed by students, produce the most adaptive learning outcomes. However, recent research paints a more complex picture of their endorsement by students (Vansteenkiste et al., 2014; Senko and Tropiano, 2016; Sommet and Elliot, 2017). Using self-determination theory (SDT; Ryan and Deci, 2017), researchers have shown that students endorse mastery goals for different reasons. Some pursue them for autonomous reasons, while others have controlled reasons. The former predict more adaptive learning outcomes (e.g., Gaudreau, 2012; Benita et al., 2014; Michou et al., 2014). We took this reasoning a step further and suggested that students are likely able to perceive their teachers as autonomy supportive or controlling when they present mastery goals in the classroom, and these different motivational styles will be related to the degree to which students internalize mastery goals and to their behavioral engagement.

### Mastery Goals: Their Optimal Outcomes and How Teachers Promote Them

The type of goals students endorse in the classroom is an important determinant of their experience, behavior, and performance (Boekaerts et al., 2006). Ames (1992) defined a mastery goal as an ambition to improve the level of competence, to develop new skills, or to achieve a sense of mastery based on self-referenced (intrapersonal) standards. Mastery goals have often been contrasted with performance goals, defined by Ames (1992) as an ambition to demonstrate competence and to perform better than others, where one’s self-worth is contingent upon one’s performance. Research anchored in achievement goal theory has found that students’ endorsement of mastery goals is related to more positive educational outcomes, such as positive emotional experiences, persistence and effort during challenging tasks, and deep learning processes (e.g., Harackiewicz et al., 2000; Levy et al., 2004; Wolters, 2004; Pekrun et al., 2006; Darnon et al., 2007; Gonida et al., 2009; Benita et al., 2014). Both types of goals can be framed either as positive outcomes that can be approached or as negative outcomes that need to be avoided (Elliot and McGregor, 2001). In this research, we only examined mastery-approach goals, and to a lesser extent performance-approach goals. We did not examine their avoidance counterparts.

Achievement goal researchers have also differentiated between the goals endorsed by students (i.e., personal achievement goals) and the goals promoted by teachers in the classroom (e.g., Ames, 1992; Kaplan et al., 2002). These researchers have advocated that teachers should promote mastery goals as a pathway to students’ endorsement of the goals and, hence, their adaptive learning.

To measure students’ endorsement of mastery goals and the practices teachers use to promote them, Midgley et al. (2000) developed the Patterns of Adaptive Learning Scales (PALS). In these scales, students’ personal mastery goals include 1) striving to develop competence and extending knowledge and understanding and 2) enjoying learning. The scales assessing teachers’ practices when presenting mastery goals include 1) accepting students’ mistakes for the sake of learning; 2) encouraging thorough learning instead of memorization; and 3) encouraging students’ enjoyment and interest. Research using PALS (Midgley et al., 2000) has demonstrated that students’ perceptions of teachers’ promotion of mastery goals are related to their own endorsement of them (e.g., Friedel et al., 2007; Matos et al., 2017).

### Controversy Around the Achievement Goal Construct

As achievement goal theory developed, controversy sprang up, especially around the definition of the term “achievement goal” (Senko et al., 2011). Traditional definitions of this term referred to it as a broad *orientation* toward competence and achievement, and it was thought to include various competence-based constructs, such as aims, reasons, and feelings (Dweck, 1986; Ames, 1992). Specifically, traditional definitions of personal mastery goals, as evident in the PALS’s items measuring the goals (Midgley et al., 2000), included both *aims*, such as developing competence, and *reasons*, such as engaging in learning activities because they are enjoyable. For example, students who endorse mastery goals were considered those who strive to develop knowledge (i.e., their aim) because they enjoy learning (i.e., their reason). Recently, Senko and Tropiano (2016) referred to these traditional models as *achievement goal-orientation models*.

At the turn of the century, however, Elliot and Thrash (2001) and Elliot and Murayama (2008) criticized these achievement goal models and their view of goals as broad orientations that include both aims and reasons. They suggested that such a broad definition lacks precision and limits the possibility of the achievement goals framework to move forward. They thus pursued a narrower and more precise definition and suggested that “goal” should be defined solely as an aim or as a *standard of competence*. By so doing, they excluded reasons or motives entirely. For example, they defined mastery goals as composed of two types of competence-related standards: intrapersonal (improving knowledge) and absolute (mastering knowledge). Senko and Tropiano (2016) referred to Elliot and colleagues’ model as the *goal standard model* of achievement goals.

Relying on Elliot and colleagues’ work, researchers have more recently noticed a problem with the goal orientation model’s broad definition of goals (for a review, see Vansteenkiste et al., 2014). This criticism argued that viewing goals as broad orientations confounds specific aims with specific reasons. For example, if students endorse the intrapersonal standard typifying mastery goals, they are also considered as those who do so because they enjoy learning. In other words, for mastery goals, the aims of self-improvement and task mastery are inseparable from reasons such as interest and enjoyment.

An emerging line of research is challenging this view, showing that if achievement goals are defined solely as aims, they may be accompanied by distinct and even opposing reasons, and this might impact their effects on various sets of outcomes (e.g., Dompnier et al., 2009; Vansteenkiste et al., 2010; Benita et al., 2014; Michou et al., 2014). Many researchers taking this approach have espoused an SDT (Ryan and Deci, 2017) perspective.

### Self-Determination Theory: Autonomy vs Control

Self-determination theory (Ryan and Deci, 2017) is an approach to motivation emphasizing the motives or reasons individuals have for their behaviors, beliefs, values, and goal pursuits. One of the tenets of SDT is the concept of *internalization*, defined as the process of taking in values and goals from external sources and transforming them into one’s own (Ryan and Deci, 2000). The theory differentiates between two broad categories of internalization of behavior or what is generally called motivation regulation: autonomous motivation, whereby individuals endorse the goals set by socialization agents and perceive them as their own, and controlled motivation, whereby individuals perceive their goals as imposed on them by external sources.

In school, controlled and autonomous motivations are manifested in students’ reasons for pursuing learning activities (Ryan and Connell, 1989). External and introjected reasons represent controlled motivation and refer to engaging in an activity because one is motivated to comply with external demands or to avoid feelings of guilt or shame. In contrast, identified and intrinsic reasons represent autonomous motivation and refer to engaging in an activity because it is personally meaningful or simply because it is fun.

Self-determination theory researchers have also examined factors in the social environment that either facilitate or diminish internalization of behaviors and goals (Ryan and Deci, 2017). In the classroom setting, autonomy-supportive teachers take the students’ perspective, act in ways that encourage choice and self-initiation, and provide meaningful rationales and relevance (Reeve and Jang, 2006; Reeve, 2016). Meanwhile, controlling teachers tend to use rewards, deadlines, threats, and pressuring language to control students’ behavior (Reeve, 2009). These different teaching methods (autonomy support vs control) are most commonly measured by students’ perceptions of teacher behaviors (Reeve, 2016).

Researchers are often interested in the relations between teacher autonomy support and control and students’ functioning, as manifested by their *engagement* versus *disengagement*. Engagement refers to the extent of a student’s active involvement in a learning activity (Reeve, 2012). It is a multidimensional construct, involving behavioral, cognitive, and emotional components (Ben-Eliyahu et al., 2018). In the present paper, we focused on *behavioral engagement* (vs disengagement). This concept refers to students’ involvement in the learning activity, or lack thereof, in terms of attention, effort, and persistence (Skinner et al., 2009). Students who are behaviorally engaged tend to participate in class, listen to their teacher, follow instructions, and put effort into schoolwork. This, therefore, is an observable manifestation of internalization or an indicator of the extent to which children are willing to endorse a teacher’s goals and cooperate.

Research consistently shows that students’ perception of autonomy-supportive teaching positively predicts students’ behavioral engagement (Assor et al., 2002; Jang et al., 2010, 2012; Matos et al., 2018), while students’ perception of controlling teaching predicts their behavioral disengagement (e.g., Assor et al., 2005; Jang et al., 2016). Research also suggests that the effects of teacher autonomy support and control on engagement are mediated by students’ reasons for learning. Students perceiving their teachers as autonomy supportive are more likely to be engaged in learning because they fully internalize their teacher’s goals (e.g., Hagger et al., 2015; Kaplan, 2018). Students perceiving their teachers as controlling are less likely to be engaged because they only partially internalize their teacher’s goals (e.g., Assor et al., 2005; Soenens et al., 2012).

### The Goal-Complex Model of Achievement Goals

Recent developments in the achievement goal framework paved the way for its integration with SDT. Senko and Tropiano (2016) suggested that this integration created a unique achievement goal model: the *goal-complex model of achievement goals*. In this model, a certain aim (an achievement goal, e.g., a mastery goal) can be pursued despite opposing underlying reasons (autonomous vs controlled). For example, some students can strive to improve knowledge (endorse mastery goals) because they truly acknowledge the importance of doing so (e.g., “It is important for me to improve my math abilities), while others feel compelled to do so (e.g., “I must improve my math abilities”). Such different goal-reason combinations are goal complexes (Elliot, 1999, 2005). This model is similar to the goal standards model (and different from the goal orientation model) in that it defines goals as standards of competence, not as orientations. However, it is different from the goal standards model in that it also assesses students’ reasons for learning, not just their goals. Because the goal-complex model does not bind specific goals with specific reasons, it enables greater flexibility. Senko (2016) claimed that this model both reconciles the rival conceptualizations of the achievement goal model and better accounts for the data (e.g., Senko and Tropiano, 2016; Sommet and Elliot, 2017).

A growing body of literature on mastery goals now espouses the goal-complex model and demonstrates that students can pursue mastery goals for both autonomous and controlled reasons (for a review, see Vansteenkiste et al., 2014). Such research has consistently demonstrated that mastery goals pursued for autonomous reasons predict better learning outcomes than those pursued for controlled reasons. Several studies (Michou et al., 2014, 2016; Sommet and Elliot, 2017) found that autonomous reasons for mastery goals predict positive learning outcomes such as deep learning strategies, effort expenditure, and enjoyment, beyond the effect of the goals themselves. Other studies explored how the interaction between mastery goals and their underlying reasons predict learning outcomes. For instance, Gaudreau (2012) found that mastery goals predicted students’ grades and academic satisfaction only to the extent to which they were endorsed for autonomous reasons, and the goals’ negative relations with academic anxiety were evident only when the reasons underlying them were non-controlled. Similarly, Benita et al. (2014) found that mastery goals predicted behavioral and emotional engagement more when accompanied by a higher (rather than lower) sense of autonomy.

We suggest that the same logic applies to the promotion of mastery goals by teachers. In other words, just as students can endorse mastery goals with underlying autonomous and controlled reasons, teachers can promote the goals using autonomy supportive or controlling practices. For instance, some teachers can promote mastery goals in the classroom but use controlling language when doing so. Such teachers may forcefully demand that students master the class material. Other teachers are likely to be autonomy supportive when presenting mastery goals. For example, they may offer a meaningful rationale explaining why mastering the material is important and acknowledging students’ feelings if they struggle.

The assumption that mastery goals can be promoted in both autonomy supportive and controlling ways has been recently demonstrated in several experimental studies (Spray et al., 2006; Benita et al., 2014, 2017; Mulvenna et al., 2020). In these studies, both goals (mastery vs performance) and communication styles (autonomy supportive vs controlling) were manipulated in the lab. The researchers found that the communication of mastery goals in autonomy supportive (vs controlling) ways yielded higher task enjoyment (Spray et al., 2006; Benita et al., 2014), less tension (Benita et al., 2014, 2017), greater perceived choice (Benita et al., 2014), more free-choice behavior (Spray et al., 2006), and better performance (Spray et al., 2006; Benita et al., 2017; Mulvenna et al., 2020).

However, these studies did not explore the issue in the classroom context, the place where mastery goals are typically promoted and endorsed. Rather, they used contrived experimental tasks. Nor did they consider mastery goals’ internalization; in fact, no study has tested whether students differentially internalize and endorse mastery goals as a function of their perception of their teacher as autonomy supportive or controlling when presenting mastery goals. The present research addressed both lacunae.

### The Present Research

This research had two complementary goals. The first was to demonstrate that students can perceive their teachers as oriented toward autonomy support or control when they present mastery goals. The second was to test a model suggesting students are more likely to internalize mastery goals when teachers promote them in autonomy supportive (vs controlling) ways and that promoting the goals in autonomy supportive ways yields benefits in terms of students’ engagement.

To attain the first goal, we developed a new instrument in Study 1. Currently, the sole extant measure assessing teachers’ promotion of mastery goal in the classroom is PALS (Midgley et al., 2000). However, because the PALS confounds aims and reasons, it captures only one type of mastery goal complex, the autonomy support-mastery goal complex. We suggest that the PALS misses an important part of picture, that is, the possibility that teachers can promote students using controlling language. Therefore, in Study 1 we assessed the validity and reliability of a tool assessing students’ perceptions of their teachers’ motivational styles (autonomy supportive vs controlling) when they present mastery goals. To attain the second goal, in Study 2 we used a multilevel approach to examine the relations between students’ perceptions of teachers’ autonomy support vs control when presenting mastery goals, students’ own endorsement of the goals, students’ engagement and disengagement, and students’ pursuit of learning activities for autonomous vs controlled reasons.

This research was conducted in homeroom classes in Israel. In this country, especially in elementary and middle schools, students spend most of their time in their homeroom; various subject teachers enter the room to teach them. Some teachers, in addition to their role as subject teachers, are also homeroom teachers. Homeroom teachers teach their homeroom students one or two subjects and are responsible for all scholastic issues involving the student. This includes keeping in regular touch with subject teachers, tracking students’ progress and mastery of all subjects, assessing their overall academic performance, and communicating this to students and parents. Thus, they play a crucial role in communicating achievement goals to students. In addition, they are required to deal with the social cohesion of the class, meeting needs that are not strictly academic (Razer et al., 2015). Given their crucial social and academic role, we assumed that the extent to which such teachers are autonomy supportive (vs controlling), especially when they set students mastery goals, is likely to play a crucial role in influencing students’ internalization of these goals and their subsequent class engagement.

## Study 1

Our first aim was to demonstrate that students perceive their teachers as oriented toward autonomy support or control when they present mastery goals. To do so, we developed a new vignette-based instrument, which offers respondents authentic situations in which teachers present mastery goals to students and depicts two ways teachers are likely to promote the goals, autonomy supportive and controlling. The use of vignettes is a common way to assess teachers’ autonomy supportive or controlling motivational styles (Deci et al., 1981; Aelterman et al., 2019). Because teachers do not present mastery goals all the time, the use of scenarios propels students to generate a clear but imagined representation of their teachers’ promotion of mastery goals in autonomy-supportive and controlling ways.

The specific aim of Study 1 was to test the new scale’s validity and reliability. To establish the scale’s validity, we accepted Hughes’s (2018) contention that a scale’s validity can be determined by two complementary aspects, its accuracy and appropriateness. *Accuracy* refers to the closeness of a measurement to its correct value, namely, whether it measures what it purports to measure. We established the instrument’s accuracy by assessing its content validity, structure validity (factor structure), and stability across groups (male vs female students). We hypothesized that the scale would yield two distinct constructs, one for autonomy-supportive presentation of mastery goals and one for controlling presentation, as evidenced by the content analysis and factor structure (Hypothesis 1). Second, we expected the scale would not vary between genders (Hypothesis 2). *Appropriateness* refers to whether the scale is suitable or appropriate to use in a given situation. We established the scale’s appropriateness by assessing its discriminant and criterion validity.

First, to assess the scale’s discriminant validity, we had to differentiate it from similar but distinct constructs. The construct we explored was students’ autonomous vs controlled reasons for pursuing mastery goals. This was done to support the assumption that students’ responses represent teachers’ actual behaviors, and not projections of students’ own autonomous or controlled motivations for pursuing mastery goals. Thus, we used confirmatory factor analysis (CFA) and designed a four-factor model that includes the following latent variables: teachers’ autonomy support vs control when presenting mastery goals and students’ autonomous vs controlled reasons to endorse mastery goals. Hypothesis 3 was that this model would fit the data better then alternative models in which perceptions of autonomy support vs control for mastery goals tapped the same constructs as students’ autonomous vs controlled reasons.

Next, to test the measure’s criterion validity, we explored how students’ perceptions of teachers’ autonomy support vs control when presenting mastery goals correlated with a range of external variables, including students’ personal achievement goals (mastery vs performance), students’ autonomous vs controlled reasons to pursue mastery goals, students’ engagement and disengagement, and students’ vitality (an indicator of well-being). Hypothesis 4 was that students’ perceptions of autonomy support for mastery goals would be positively related to adaptive outcomes (students’ personal mastery goals, autonomous reasons to pursue mastery goals, engagement, and vitality). We expected that their perception of teachers’ controlling presentation of mastery goals would positively correlate with maladaptive outcomes (students’ personal performance goals and controlled reasons to pursue mastery goals).

### Method

#### Participants and Procedures

Two independent samples of students were used throughout the two phases of the study. Sample 1 involved 113 Israeli Jewish^1^ students in grade 7 (52% girls; mean age = 12.29 years) from four classes in one school, with an average of 28.3 students per class. Sample 2 involved 204 Israeli Jewish students in grades 7–9 (57% girls; mean age = 13.32 years) and their homeroom teachers from eight classes in one school, with an average of 25.5 students per class. Both schools served middle-class families, as classified by the Israeli Ministry of Education. Students in Sample 1 participated in a pilot study and filled in only the new instrument. Students in Sample 2 filled in the new instrument, as well as measures purported to establish the scale’s discriminant and criterion validity. Students’ questionnaires were administered by trained research assistants. For both samples, students completed the questionnaires in class during one session. The teacher was not present in the classroom. All questionnaires were administered in Hebrew, students’ and teachers’ mother tongue. Our procedure followed the ethical guidelines of Israel’s Ministry of Education. The research was authorized, and parents provided their consent according to the formal guidelines. Research assistants instructed students to refer to their homeroom teacher’s class when answering the entire questionnaire packet. Therefore, students’ reports referred to four and eight unique teachers in the pilot and the main studies, respectively. Research assistants administered the teachers’ questionnaires and collected them in sealed envelopes 1 week later. Teachers rated each students’ behavioral engagement in the classroom. All eight teachers returned the questionnaires.

#### Measures

All questionnaires were on a Likert-type scale, ranging from *not at all true* (1) to *very true* (6), except where indicated. Items in each scale were presented in a mixed order.

##### Students’ perceptions of teachers’ autonomy support vs control when presenting mastery goals

This vignette-based instrument was developed for this study in Hebrew. The final instrument is presented in Table 1 in English. Translation to English and back translation to Hebrew was done by an expert bilingual translator.

**TABLE 1:** Pilot: vignettes, items, and factor loadings for measure assessing students’ perceptions of teacher autonomy support vs control for mastery goals.

**Vignettes and Items**	**Component 1**	**Component 2**
Vignette 1: Your teacher has seen your achievements and thinks you could improve your knowledge in the subjects she teaches. Based on your experience with your teacher, how would she instruct you to improve your knowledge in the subject she teaches?		
She will share with you why she thinks it’s important for you to improve	0.66	
She will explain how improving could be useful for you	0.76	
She will encourage you to choose the right way for you to improve	0.77	
She will put pressure on you to improve		0.81
She will make you feel like you must improve		0.68
She will try to force you to improve		0.75
Vignette 2: The Ministry of Education published the subject matter to be mastered for an international exam in math and science. Based on your experience with your teacher, how would she instruct you to master those subjects?		
She will share with you why she thinks it’s important for you to master the material	0.76	
She will explain how mastering the material could be useful for you	0.74	
She will encourage you to choose the right way for you to master the material	0.79	
She will put pressure on you to master the material		0.81
She will make you feel like you must master the material		0.56
She will try to force you to master the material		0.82

Following previous tools assessing teacher autonomy support and control (Deci et al., 1981), we generated two short vignettes describing how the teacher promotes mastery goals in the classroom (see Table 1). Each vignette was followed by 6 “autonomy support” items and 6 “control” items. Each item had a parallel item (i.e., similar items) in the two vignettes and has been modified to the vignette’s content, making a total of 12 pairs of items. We based our items on existing scales assessing teacher autonomy support (learning climate questionnaire; Williams and Deci, 1996) and directly controlling teacher behaviors (controlling teacher questionnaire; Jang et al., 2009). To further establish the instrument’s content validity, we sent it to two experts (a teacher and a researcher) for review; when we received their comments, we revised the items and vignettes accordingly.

Then, we administered the new instrument to the pilot’s sample and examined its factor structure using principal component exploratory factor analysis (EFA). The initial solution yielded four components. Nineteen items were loaded on the first two components. The first component (eigenvalue = 6.70) included 10 items (five items per each vignette) tapping students’ perception of teachers’ control. The second component (eigenvalue = 4.71) included nine items (four and five items for the first and second vignette, respectively), tapping perceptions of teachers’ autonomy support. All factor loadings were above 0.47. The remaining five items tapped another two components, with eigenvalues of 1.50 and 1.21. With the aim of reducing the number of items to six per vignette (three for autonomy support and three for control), we first removed the five items loaded on the third and fourth components and another item which did not have a counterpart in both vignettes. We then selected six pairs of items, those with the highest factor loadings (above 0.66). The final list of items is presented in Table 1. We conducted another principal component EFA this time with the 12 remaining items. This analysis yielded two components, one for perceptions of teachers’ autonomy support (eigenvalue = 4.42) and the other for perceptions of teachers’ control (eigenvalue = 2.36). These two components explained together 56.48% of the variance. Factor loading for each item is presented in Table 1. Cronbach’s alpha reliabilities were 0.84 and 0.85 for the items tapping teachers’ autonomy support and control, respectively.

After the pilot, we administered the new instrument to the main sample of students and conducted a series of CFAs for a more rigorous examination of the instrument’s factor structure. Because students were nested in classes, we adjusted for the hierarchical nature of the data by using class as the “cluster” variable in the “Type = Complex” method in Mplus 7.11 (Muthén and Muthén, 2012). To establish the metric equivalence of the scales across genders, we conducted a multigroup CFA. This analysis tested whether the item loadings on the constructs they were assumed to capture were equivalent across genders. To assess equivalence, we compared a model in which the item loadings on each latent construct were constrained to be equal across genders, with an unconstrained baseline model in which these item loadings were allowed to vary across genders.

Next, we assessed the measure’s discriminant analysis by designing a four-factor model in CFA, with the following latent variables: students’ perceptions of teacher autonomy support vs control in presentation of mastery goals and students’ autonomous vs controlled reasons to pursue mastery goals. In the context of discriminant validity, CFA typically involves the comparison of two measurement models – an unconstrained model and a constrained model (Shaffer et al., 2016). In the unconstrained model, two latent variables that represent two conceptually similar constructs are allowed to freely covary with each other. In the constrained model, the covariance of these two latent variables is set to equal 1.0. We thus compared our baseline four-factor model with a constrained model, in which we constrained to 1.0 the covariances between the latent variables of perceptions of teachers as autonomy-supportive vs controlling when presenting mastery goals and students’ autonomous vs controlled reasons to pursue the goals, respectively. Finally, we tested the measure’s criterion validity by exploring its relations with other variables.

For all models, our estimation model was maximum likelihood with robust standard errors (MLR). Model fit was assessed using the root mean square error of approximation (RMSEA), the standardized root-mean-square residual (SRMR), and the comparative fit index (CFI). Our cutoff criteria followed Hu and Bentler’s (1999) recommendations: CFI > 0.95, RMSEA < 0.06, and SRMR < 0.08. To compare models, we used the Satorra–Bentler scaled chi-square difference test (Satorra and Bentler, 2010).

##### Students’ personal achievement goals

We used the revised Achievement Goal Questionnaire (AGQ-R; Elliot and Murayama, 2008) to assess students’ mastery goals (three items; e.g., “My aim is to completely master the material presented in this class”) and performance goals (three items; e.g., “My aim is to outperform others in this class”), adapted to the school context by Benita et al. (2014). Cronbach’s alpha was 0.89 and 0.88 for mastery and performance goals, respectively.

##### Underlying reasons for achievement goals

This scale was developed by Vansteenkiste et al. (2010) and was previously used among high school students by Michou et al. (2014). The scale was translated into Hebrew and back translated to English by a bilingual expert translator. We first presented participants with one item from the revised AGQ-R (Elliot and Murayama, 2008), assessing the extent to which they adopted mastery goals. If students’ response was 4 and above (indicating they pursued the goal), we asked them to indicate their reasons for pursuing it. Three items assessed autonomous reasons (e.g., “Because this is an important goal to me”). Three items assessed controlled reasons (e.g., “Because I would feel guilty if I didn’t do so”). One hundred and ninety-seven students reported adopting mastery goals (responded 4 or more on the AGQ-R’s mastery goals items) and thus completed the items on reasons for endorsing the goals. Cronbach’s alpha was 0.78 and 0.70 for autonomous and controlled reasons, respectively.

##### Students’ subjective vitality

We used the Hebrew version of the Subjective Vitality Scale (Ryan and Frederick, 1997), and we adjusted it to the homeroom teacher’s classes context (five items; e.g., I feel alive and vital in my teacher’s lessons). Cronbach’s alpha was 0.88.

##### Teachers’ reports of students’ behavioral engagement

This measure, developed by Assor et al. (2005), assesses students’ behavioral engagement (three items; e.g., “This student shows persistence as she/he works on assignments”). Homeroom teachers filled in the scale for each of their homeroom students. Teachers responded on a Likert-type scale ranging from *not at all true* (1) to *very true* (5). Cronbach’s alpha was 0.94.

### Results

#### Instrument’s Accuracy

**FIGURE 1:**
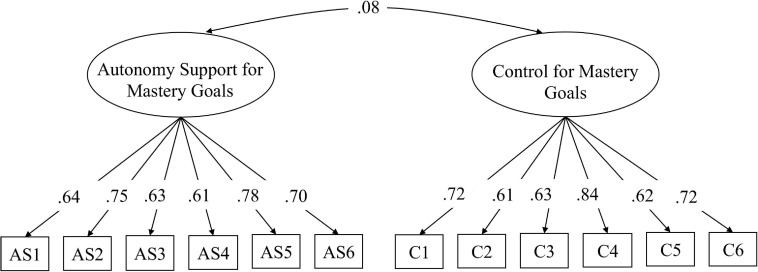
Study 1. Construct validity of the measure assessing students’ perceptions of teacher autonomy support vs control for mastery goals.

The instrument’s content validity was established by relying on previous scales assessing autonomy support and controlling teacher behaviors and by the process of expert review described above. To explore Hypothesis 1, we examined the scale’s structure validity using CFA. The results are presented in Figure 1. As expected, this analysis yielded adequate fit indices, χ^2^_(__83__)_ = 83.13, *p* < 0.003; RMSEA = 0.06; SRMR = 0.06; CFI = 0.95. As seen in the figure, each item was loaded on its respective factor. Then, to examine Hypothesis 2 (metric equivalence across genders), we compared the fit of the constrained and unconstrained models. Fit indices of both models were satisfactory (χ^2^_(__97__)_ = 140.61, *p* < 0.001, RMSEA = 0.07, SRMR = 0.07, CFI = 0.95 for the unconstrained model; χ^2^_(__108__)_ = 149.67, *p* < 0.005, RMSEA = 0.06, SRMR = 0.08, CFI = 0.95 for the constrained model). The Satorra–Bentler scaled chi-square difference test (Satorra and Bentler, 2010) yielded non-significant difference between models (TRd_(__11__)_ = 9.73, *p* > 0.05), indicating an acceptable factor structure that did not significantly vary across genders. Cronbach’s alpha reliabilities were 0.85 for both the autonomy support and the control items. Based on the results, we created the two scales (perceptions of autonomy support and perceptions of control in presentation of mastery goals), by calculating the means of the respective items. The two scales were uncorrelated (*r* = 0.07, ns).

#### Instrument’s Appropriateness

We then examined the scale’s discriminant validity (Hypothesis 3). As noted above, the baseline model was designed to include four distinct factors. As seen in Table 2, this model yielded adequate fit indices. We compared this model with an alternative two-factor model. As the table shows, model fit for the baseline model was superior to this model. The Satorra–Bentler chi square difference test indicated that chi square differences between the baseline model and the alternative model were significant. Thus, the four-factor model, in which perceptions of teachers as autonomy supportive vs controlling in their presentation of mastery goals and students’ reasons underlying mastery goals represented distinct constructs, yielded the best fit for the data.

**TABLE 2:** Study 1: Discriminant validity: comparison of the hypothesized model and the alternative model.

	**χ ^2^**	***Df***	**CFI**	**RMSEA**	**SRMR**	**Satorra–Bentler **Δ**χ ^2^ (TRd)**
Model						
Four-factor model (baseline model)	167.21**	125	0.96	0.04	0.07	
Reasons-only model	376.36**	130	0.77	0.09	0.10	149.95**

CFI, comparative fit index; RMSEA, root-mean-square error of approximation; SRMR, standardized root mean square residual. **p < 0.01.

To test the instrument’s criterion validity (Hypothesis 4), we examined its nomological network. Table 3 presents the descriptive statistics and intercorrelations between the study variables. As seen, all correlations were in the expected direction. Of note, perceptions of teachers’ autonomy support for mastery goals in their presentation of these goals were positively correlated with students’ personal mastery goals, students’ vitality, and teachers’ reports of behavioral engagement. Perceptions of teachers’ control when presenting mastery goals were positively associated students’ personal performance goals and with students’ controlled endorsement of mastery goals.

**TABLE 3:** Study 1: descriptive statistics, reliabilities, and intercorrelations between the study variables.

	**Variable**	**1**	**2**	**3**	**4**	**5**	**6**	**7**	**8**
1	Autonomy support for mastery goals	−							
2	Control for mastery goals	0.07	−						
3	Students’ mastery goals	0.46**	–0.12	−					
4	Students’ performance goals	0.13	0.41**	0.14*	−				
5	Autonomous mastery goals	0.33**	0.09	0.34**	0.20**	−			
6	Controlled mastery goals	–0.14	0.39**	−0.17*	0.39**	0.13	−		
7	Vitality	0.33**	0.03	0.15*	10	0.31**	0.00	−	
8	Behavioral engagement (teachers’ report)	0.21**	–0.05	0.19**	0.01	0.29**	–0.10	0.45**	−

*p < 0.05, **p < 0.01.

### Brief Discussion

The results of Study 1 supported the hypotheses. Specifically, the results supported the accuracy of the scale assessing students’ perception of teachers’ autonomy support and control when they set mastery goals, as observed by the scale’s construct validity, its equivalence across genders, and its reliability. The results also supported its appropriateness. Discriminant validity results showed it yielded a unique construct, distinct from measures of students’ reasons for pursuing the goals. Criterion validity results indicated that each construct (perceptions of autonomy support vs control when presenting mastery goals) predicted distinct, and even opposing, outcomes. Therefore, there was good evidence supportive of the validity and reliability of the instrument, allowing us to continue using this instrument in the next study.

An important finding of Study 1 was that students’ perceptions of their teachers as autonomy supportive, but not controlling, when presenting mastery goals were positively associated with students’ personal mastery goals and with their autonomous reasons to endorse the goals. Thus, students who perceived their teachers as autonomy supportive when presenting mastery goals were more likely to internalize the goals. In Study 2, we took a further step by exploring these relations. In this study, we used a larger sample, enabling us to conduct a multilevel analysis (Raudenbush and Bryk, 2002). The advantage of multilevel analysis is that it divides the variance in students’ reports, specifically those on teachers’ autonomy support vs control when presenting mastery goals, to within- and between-classroom levels. The former type of variance stems from differences between students’ individual perceptions across different classrooms. The latter stems from differences between different classrooms and reflects the agreement among students in a given classroom on their perception of their teacher. It thus represents teachers’ observable behavior more accurately. Specifically, the use of a multilevel design enabled us to explore whether in classrooms where teachers are perceived as autonomy supportive (vs controlling) when presenting mastery goals, students are more likely to endorse the goals and to be more engaged. Our first hypothesis was that at both individual and classroom levels, perceptions of teachers as autonomy supportive when presenting mastery goals would be related to students’ personal mastery goals, as well as to their engagement. We also expected that perceptions of teachers as controlling when presenting the goals would be related to students’ disengagement at both levels.

Our second goal in this study was to explore the mechanisms explaining why students’ perceptions of their teachers’ autonomy support vs control when presenting mastery goals are differentially related to students’ mastery goals endorsement and engagement. Following previous studies (e.g., Hagger et al., 2015), we hypothesized the increased likelihood to endorse the goals presented by teachers would be explained by such students’ tendencies to internalize the importance of learning activities. Thus, our second hypothesis was that autonomous reasons for learning would mediate the relations of students’ perceptions of teachers as autonomy supportive when presenting mastery goals, the goals’ endorsement, and students’ engagement. We also hypothesized that controlled reasons for learning would mediate the relations between perceptions of teachers as controlling in their presentation of mastery goals and disengagement.

## Study 2

### Method

#### Participants and Procedure

Participants were 1,303 Israeli Jewish students in grades 7–9 (50% girls; mean age = 13.29 years) from 60 classes in five schools serving middle-class families. The average class size was 20.83. Students completed the questionnaires in class during one session lasting about 30 min. The teacher was not present in the classroom. All questionnaires were administered in Hebrew, students’ mother tongue. Our procedure followed the ethical guidelines of Israel’s Ministry of Education. The research was authorized, and parents provided their consent according to the formal guidelines. Research assistants instructed students to refer to their homeroom teacher’s class when answering the entire questionnaire packet. Therefore, students’ reports referred to 60 unique teachers. In this study, we lacked the option of collecting data from teachers due to technical limitations. Therefore, all measures were reported by students.

#### Measures

All questionnaires were on a Likert-type scale, ranging from *not at all true* (1) to *very true* (6). Items in each scale were presented in a mixed order. Cronbach’s alpha coefficients for the scales are presented in Table 3. All variables were created by averaging items tapping their respective scales.

##### Students’ perceptions of teachers’ autonomy support vs control when presenting mastery goals

This was the measure validated in Study 1.

##### Students’ mastery goals

As in Study 1, we used the AGQ-R (Elliot and Murayama, 2008) to assess students’ mastery goals (three items).

##### Students’ autonomous vs controlled motivation for learning

Four subscales were taken from Ryan and Connell’s (1989) scale of perceived locus of causality for the academic domain, adapted and validated for Jewish Israeli elementary students by Assor et al. (2005). Students were asked their reasons for participating in several learning-related behaviors: doing homework, participating in class-work, and trying to do well in school. Each behavior was followed by items assessing the degree to which they pursued it. We examined two types of controlled reasons: external (e.g., “I prepare homework because I don’t want the teacher to be mad at me”) and introjected (e.g., “I try to answer hard questions in class because I will be ashamed of myself if I don’t”). We also examined two types of autonomous reasons: identified (e.g., “I try to do well in school because it’s important to me”) and intrinsic (e.g., “I do homework because it’s fun”). In the Israeli version, each type of motivation is assessed by four items. The controlled motivation score was a composite of external and introjected scales, and the autonomous motivation score was a composite of intrinsic and identified scales.

##### Behavioral engagement

This 4-item scale was taken from Benita et al. (2014) and used to assess students’ adaptive functioning in relation to class engagement (e.g., “I do more than what I am required when I study the subjects taught by teacher.”).

##### Behavioral disengagement

This 3-item scale was taken from Assor et al. (2005) and used to assess students’ behavioral disengagement (e.g., “I do not even try to succeed in the subjects the teacher teaches”).

### Results

#### Plan of analysis

We first calculated correlations between the study variables. Then, to test our mediation hypothesis, we used the syntax provided by Preacher et al. (2010), using Mplus version 7.11 (Muthén and Muthén, 2012) to conduct multilevel structural equation modeling (SEM). MSEM overcomes the problems posed by traditional multilevel mediation analysis by providing a more accurate estimation of indirect effects by decomposing the variance into two (within-level and between-level) components (Muthén and Asparouhov, 2011). As all of the constructs in our model were assessed at Level 1 (the student level), they contained both within- and between-class variance. This procedure enabled us to examine the hypothesized mediation in both levels. We thus assessed a lower level mediation model (i.e., a 1–1–1 mediation model; Krull and Mackinnon, 1999).

Indirect effects should be based on bootstrapped standard errors (MacKinnon et al., 2002), but Mplus 7.11 does not allow bootstrapping with multilevel analyses. Therefore, we tested indirect effects in our model with the (robust) maximum likelihood estimates, with standard errors estimated via the delta method (the Mplus default).

#### Preliminary Analysis

Table 4 presents the descriptive statistics for each scale and their intercorrelations. Of note, perceptions of autonomy support and control in goal presentation were both positively related with autonomous motivation, mastery goals, and behavioral engagement, but the relations with autonomy support were stronger. Perceptions of autonomy support in mastery goal presentation were also negatively related with disengagement, but perceptions of control were not. Finally, perceptions of autonomy support and control were both positively related with controlled motivation, but the relations were stronger for the latter.

**TABLE 4:** Study 2: Descriptive Statistics, Reliabilities, and Intercorrelations of Study Variables.

	**Variable**	***M***	***SD***	**Cronbach’s α**	**ICC**	**1**	**2**	**3**	**4**	**5**	**6**	**7**
1	Autonomy support for mastery goals	4.59	1.02	0.84	12%	–						
2	Control for mastery goals	3.31	1.14	0.83	5%	0.27**	–					
3	Autonomous motivation	3.38	0.99	0.70	8%	0.42**	0.17**	–				
4	Controlled motivation	2.94	0.98	0.73	2%	0.10**	0.28**	0.37**	–			
5	Mastery goals	5.12	0.96	0.87	4%	0.34**	0.13**	0.44**	0.16**	–		
6	Behavioral engagement	3.48	1.16	0.76	13%	0.44**	0.18**	0.59**	0.24**	0.36**	–	
7	Behavioral disengagement	2.09	1.17	0.81	8%	−0.25**	0.04	−27**	0.04	−0.32**	−0.22**	–

*p < 0.05, **p < 0.01.

Before continuing to the multilevel models, we calculated the intraclass correlation (ICC) for our variables to determine the proportion of the total variance due to differences between classes. Table 4 presents the ICCs. With the exception of the controlled motivation scale, between-group variance for all measures was significant at *p* < 0.05. Following LeBreton and Senter (2008), we considered an ICC value of 0.01 a small effect, 0.10 a medium effect, and 0.25 a large effect. Overall, the ICC’s effect size was small-to-medium. The modest ICC measures for the mediator and outcome variables supported a 1–1–1 model in which mediation was assessed at both within- and between-class levels. For perceptions of autonomy support and control in the presentation of mastery goals, the significant between-classroom variance supported the assumption that, at least partly, but more for autonomy support, these reports reflected agreement among students on their particular teacher’s use of autonomy support or control when presenting mastery goals.

#### Primary Analysis

**FIGURE 2:**
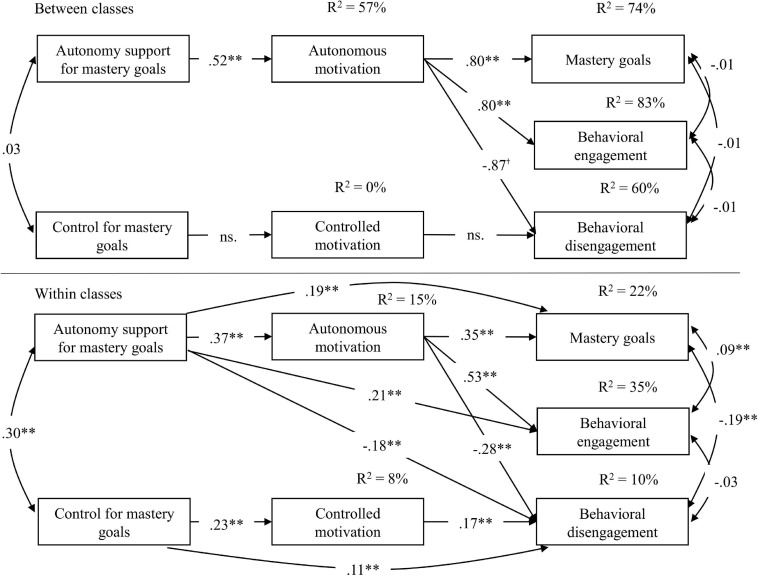
Study 2. 1–1–1 multilevel mediation model. For the sake of clarity, non-significant paths are not shown, except where indicated. SP-ASMG, students’ perceptions of teachers’ autonomy support for mastery goals; SP-CMG, students’ perceptions of teachers’ control for mastery goals. ^†^*p* < 0.10; **p < 0.01.

Results for the multilevel SEM model are presented in Figure 2. Table 5 gives the indirect effects. At the within-class level, perceptions of teachers’ autonomy support when presenting mastery goals predicted students’ personal mastery goals and behavioral engagement and negatively predicted disengagement, both directly and through autonomous motivation. Perceptions of teachers’ control when presenting mastery goals positively predicted disengagement, both directly and through autonomous motivation. As Table 5 shows, these indirect effects were significant. The significance of the direct effects indicated partial mediation. At the between-class level, perceptions of autonomy support in the presentation of mastery goals predicted the endorsement of mastery goals and behavioral engagement through autonomous motivation. Both indirect effects were significant. Unlike the within-class level, the indirect effect of perceptions of teachers’ autonomy support and control when presenting mastery goals on disengagement through autonomous and controlled motivations were non-significant.

**TABLE 5:** Study 2: indirect effects on study variables.

				**95% CI**	
				
**Indirect effect**	**Estimate**	**SE**	***P*-value**	**LL**	**UL**
**Within**					
ASMG → Autonomous reasons → Mastery goals	0.13	0.02	0.000	0.11	0.16
ASMG → Autonomous reasons → Engagement	0.20	0.02	0.000	0.16	0.24
ASMG → Autonomous reasons → Disengagement	–0.11	0.02	0.000	–0.14	–0.07
CMG → Controlled reasons → Mastery goals	–0.01	0.01	0.332	–0.01	0.01
CMG → Controlled reasons → Engagement	0.01	0.01	0.144	–0.01	0.02
CMG → Controlled reasons → Disengagement	0.04	0.01	0.002	0.02	0.06
**Between**					
ASMG → Autonomous reasons → Mastery goals	0.41	0.19	0.032	0.10	0.73
ASMG → Autonomous reasons → Engagement	0.50	0.21	0.017	0.15	0.84
ASMG → Autonomous reasons → Disengagement	–0.45	0.29	0.115	–0.92	0.02
CMG → Controlled reasons → Mastery goals	–0.03	0.09	0.751	–0.16	0.11
CMG → Controlled reasons → Engagement	0.02	0.09	0.814	–0.13	0.18
CMG → Controlled reasons → Disengagement	0.01	0.09	0.887	–0.14	0.16

*ASMG, autonomy support for mastery goals; CMG, control for mastery goals.*

### Brief Discussion

Overall, the results of Study 2 supported our hypotheses. Students who perceived their teachers as oriented toward autonomy support when they presented mastery goals were likely to endorse mastery goals and be behaviorally engaged in learning. These relations were mediated by students’ autonomous reasons for pursuing learning activities. This effect was found at both the within- and between-classroom levels. Similar findings were found for disengagement, but only at the within-classroom level. Perceptions of teachers’ control in the presentation of mastery goals, however, predicted disengagement at the within-classroom level, mediated by controlled reasons for learning.

## General Discussion

Research anchored in achievement goal theory has consistently demonstrated that the pursuit of mastery goals is related to adaptive learning outcomes (for a review, see Senko et al., 2011). Accordingly, researchers have advocated the promotion of mastery goals in the classroom as a pathway for students’ endorsement of these goals and their subsequent engagement in learning (e.g., Ames, 1992; Kaplan et al., 2002). The present research used the modified definition of mastery goals offered by Elliot and Thrash (2001), Elliot and Murayama (2008) and suggested that teachers can be perceived as either autonomy supportive or controlling when presenting them, but only autonomy support predicts the goals’ internalization and adaptive learning outcomes.

### Theoretical Implications

Past conceptualizations of the goals teachers promote in the classroom (e.g., Midgley et al., 2000) confounded the promotion of mastery goals with autonomy support. Our results indicate that when the goals are defined solely as aims (Elliot and Thrash, 2001), the two are not confounded. Teachers can be perceived as either autonomy supportive or controlling when they present mastery goals, and these perceptions are differentially related to the goals’ endorsement by students and to students’ engagement.

The research follows and extends the goal-complex model of achievement motivation (Vansteenkiste et al., 2014; Senko and Tropiano, 2016). The goal-complex model of achievement goals has emerged in recent years as a prominent framework within the achievement goal framework. By incorporating concepts from SDT into achievement goal theory, research based on this model consistently shows that autonomous achievement goal pursuit predicts more adaptive outcomes than controlled achievement goal pursuit. While previous explorations in the school context focused solely on students’ reasons to endorse the goals (e.g., Vansteenkiste et al., 2010; Michou et al., 2014), we extended the model to include students’ perceptions of teachers’ autonomy support vs control when presenting the goals. In line with the predictions of the model, perceptions of the former predicted positive outcomes, including students’ mastery goal endorsement, autonomous reasons to pursue mastery goals and engagement. Meanwhile, perceptions of teachers’ control predicted maladaptive outcomes, such as the endorsement of performance goals instead of mastery goals, controlled endorsement of mastery goals, and controlled motivation generally. These findings stress the importance of exploring different goal–reason combinations, or goal complexes, and the need to extend this exploration to include the practices teachers use to promote goals.

These results support previous experimental studies on the effect of autonomy supportive and controlling practices when presenting mastery goals (Spray et al., 2006; Benita et al., 2014, 2017), extending them to the classroom context. While the outcomes measured in the experimental studies were of little relevance to the participants’ performance or experience in real-life tasks, we assessed students’ engagement in their learning activities in their natural learning environment. In addition, while the experimental studies focused solely on the effect of autonomy supportive and controlling contexts on outcomes such as engagement and intrinsic motivation, we also showed that perceptions of teachers’ autonomy support and control when presenting mastery goals are differentially related to the goals’ internalization. However, unlike the experimental research, we cannot infer causality from our cross-sectional correlational design.

We focused on mastery goals, but similar questions should be asked about performance goals. In experimental studies, Spray et al. (2006), Benita et al. (2017), and Mulvenna et al. (2020) showed that promoting performance goals in autonomy-supportive ways predicted more adaptive outcomes than promoting them in controlling ways. Thus, in the classroom, the promotion of performance goals using autonomy-supportive practices may mitigate their negative effect on outcomes. For example, in some instances, competition is inevitable. A student may need to attain better grades than others to attend a good college, and teachers are expected to encourage students to excel, often by using normative standards. Some teachers can use controlling language and induce guilt feelings to force students to endorse such goals. Other teachers can be autonomy supportive, explaining to students why it is important for them to do better than others, acknowledging their perspective, and encouraging them to state their preferences for and opinions about the goals. Whether teachers do so in an autonomy-supportive or controlling way might be related to how the goals are endorsed and to their relations with adaptive or maladaptive outcomes.

Study 2 used a multilevel framework to explore the research questions. Our findings demonstrated that teachers’ use of autonomy supportive practices when presenting mastery goals predicted most outcomes (except for disengagement) at both the within- and between-classroom levels, but their use of controlling practices predicted outcomes only at the within-classroom level. Put otherwise, students within a given classroom seemed to agree on teachers’ autonomy-supportive presentation of mastery goals, and this uniquely predicted classroom-level engagement and autonomous motivation. There was less agreement about teachers’ controlling motivating style. Note that we asked students how teachers would present the goals to them specifically, not how they would present goals to the class as a whole. It is likely that within classrooms, teachers’ controlling behaviors change from one student to another, as a function of the student’s behavior. For example, some teachers may use more controlling methods with disengaged students than engaged ones within the same classroom (e.g., Sarrazin et al., 2006). In a similar vein, researchers have long demonstrated that relations between students’ engagement and teachers’ practices are reciprocal, such that students affect teachers’ behaviors (e.g., Skinner and Belmont, 1993; Jang et al., 2016; Schuitema et al., 2016; Matos et al., 2018). Unfortunately, our cross-sectional design did not allow us to test for reciprocal relations.

There were some differences in the correlations between Study 2 and Study 1. Unlike Study 1, in Study 2, positive relations emerged between perceptions of teachers’ autonomy support and control when presenting mastery goals. In addition, in Study 2, perceptions of teachers’ use of control to present mastery goals were positively correlated with students’ personal mastery goals and engagement. These differences can be explained by the fact that in Study 1, only one school was sampled (versus five schools in Study 2). Because teacher autonomy support and control are affected by the school climate (Pelletier et al., 2002; Soenens et al., 2012), the school participating in Study 1 may have been characterized by a greater autonomy-supportive climate, where controlling practices were associated with clearly maladaptive outcomes. Future research should explore such effects at the school level.

### Limitations and Future Directions

An important limitation of our study was the use of a cross-sectional design that precluded the ability to address causal questions. Our mediation model suggested a causal chain linking teachers’ practices (autonomy support vs control) in their presentation of mastery goals to their personal autonomous or controlled reasons for endorsing them, which then differentially led to students’ engagement and goal endorsement. This causal chain is supported by previous longitudinal studies (e.g., Hagger et al., 2015), but it cannot be established by our findings. Moreover, such a causal chain precludes the equally plausible assumption that students’ behavior and motivation affect teachers’ practices (e.g., Jang et al., 2016). Our cross-sectional design cannot decide between these two possibilities, or if they occur simultaneously. Future research would do well to use a cross-lagged design with multiple measurement points for all variables.

Our cross-sectional design also prevents addressing developmental concerns. For example, research shows that in the transition to middle school many students tend to increasingly adopt performance goals at the expense of mastery goals (Midgley et al., 1995; Paulick et al., 2013). The endorsement of performance goals often takes its toll on students, as it is linked to increased anxiety and decreased well-being and engagement. An intriguing question is whether students’ endorsement of mastery goals for autonomous reasons during elementary school may act as a buffer against later performance goals adoption. If so, greater importance should be placed on teachers’ use of autonomy supportive practices when promoting mastery goals during elementary school.

Another limitation was our measure of perceived autonomy support and control in the presentation of mastery goals; the measure did not assess the extent to which teachers actually use autonomy supportive or controlling behaviors when they set the goals. Rather, it referred to how students think their teachers are likely behave in a hypothetical scenario in which they present mastery goals. In developing this tool, we followed similar questionnaires using a scenario approach to assess autonomy support or control (Deci et al., 1981; Ryan and Deci, 2000). In our view, this measure had important advantages for our research. Because teachers do not present mastery goals all the time, the use of scenarios propels students to generate a clear but imagined representation of their teachers’ promotion of mastery goals in autonomy supportive and controlling ways. Thus, although it is still likely that students’ goals shaped their perceptions of their teachers instead of vice versa, the discriminant validity results of Study 1 suggest that students differentiated between their own reasons for adopting the goals and the practices teachers used to promote the goals. In addition, the multilevel analyses of Study 2 pointed to a significant amount of agreement between students in the same classroom about their teacher’s behaviors. Nevertheless, to validate this tool, future research should explore its convergent validity by comparing it with other methods of assessing teachers’ autonomy support and control, specifically those using teacher reports (e.g., Aelterman et al., 2019) and observational methods (e.g., Reeve et al., 2004).

Another limitation is that our sample comprised Israeli middle school students so we should be cautious about generalizing the findings to students from other countries and from other class levels. Future research should extend the exploration to other countries and class levels. Another related concern is that students in this research were asked to report on their goals and teacher behaviors in different subjects. Students’ achievement goals vary differently depending on subject. Future research should take a more fine-grained look at specific subjects, such as mathematics, science, or language.

Another limitation of this research is that it focused only on behavioral engagement, which is only one aspect of the collective engagement concept. Other aspects include emotional and cognitive engagement. These three aspects combine to form the collective engagement concept, which represents a student’s entire adaptive learning experience (Ben-Eliyahu et al., 2018). To gain a deeper understanding of the effect of teachers’ autonomy support and control for mastery goals on students’ collective engagement, future research should also investigate these other aspects of engagement.

A limitation of Study 2 was that it relied solely on students’ self-reports. Such reports are susceptible to a shared-method bias. In addition, some adolescents are less likely to interpret the items as intended. Nevertheless, in Study 1, we used both students’ and teachers’ reports of engagement, and results were in the expected direction. Past research (Lee and Reeve, 2012) and the results from Study 1 show that our students’ and teachers’ reports were moderately and positively correlated, inspiring further confidence in our results and those of Study 2.

### Practical Implications

In their sharp criticism of the accountability movement in education almost 20 years ago, Midgley et al. (2001) warned against the cost of encouraging performance goals in the classroom, stating unequivocally that education systems should promote mastery goals. We agree with Midgley et al. (2001) and other researchers (e.g., Tuominen-Soini et al., 2012). We suggest that mastery goals should be supported and encouraged to promote students’ motivation, school adjustment, and well-being. However, following previous findings (e.g., Benita et al., 2014; Michou et al., 2014), we argue that not all mastery goals are equally adaptive, as some students can pursue mastery goals for controlled reasons, and this does not predict an optimal outcome.

Although our findings are preliminary and based on a cross-sectional design, they are likely to contribute to how educational systems shape their motivational climate. Our results join past research and demonstrate the benefits of autonomy-supportive teaching (e.g., Hagger et al., 2015; Jang et al., 2016). If supported by future longitudinal studies, they can inform policymakers that to promote students’ mastery goals, it is not enough to equip teachers with practices to promote the goals; it is also necessary to help them to be autonomy supportive when presenting the goals to students.

### Summary

This research joins a growing body of work demonstrating that combining achievement goal theory with SDT can further our understanding of the underpinnings of achievement motivation. It suggests that if teachers want their students to endorse mastery goals (and be more engaged), they need to use more autonomy-supportive practices and less controlling ones.

## Data Availability Statement

The datasets presented in this study can be found in online repositories. The names of the repository/repositories and accession number(s) can be found below: https://osf.io/26rvm/.

## Ethics Statement

The studies involving human participants were reviewed and approved by Israel’s Ministry of Education Ethics Committee. Written informed consent to participate in this study was provided by the participants’ legal guardian/next of kin.

## Author Contributions

MB and LM developed the instruments together, conceptualized the research questions, and wrote the manuscript together. MB collected and analyzed the data. Both authors contributed to the article and approved the submitted version.

## Conflict of Interest

The authors declare that the research was conducted in the absence of any commercial or financial relationships that could be construed as a potential conflict of interest.

## Footnotes

1 In the Israeli school system, Jewish and Arab students attend two separate public school systems, each operating in a different language and catering to culture-specific needs of individuals who mostly live in ethnically homogeneous and separate communities.

## References

[B1] Aelterman N. Vansteenkiste M. Haerens L. Soenens B. Fontaine J. R. J. Reeve J. (2019). Toward an integrative and fine-grained insight in motivating and demotivating teaching styles: the merits of a circumplex approach. *J. Educ. Psychol.* 111 497–521. 10.1037/edu0000293

[B2] Ames C. (1992). Classrooms: goals, structures, and student motivation. *J. Educ. Psychol.* 84 261–271. 10.1037/0022-0663.84.3.261

[B3] Assor A. Kaplan H. Kanat-Maymon Y. Roth G. (2005). Directly controlling teacher behaviors as predictors of poor motivation and engagement in girls and boys: the role of anger and anxiety. *Learn. Instruct.* 15 397–413. 10.1016/j.learninstruc.2005.07.008

[B4] Assor A. Kaplan H. Roth G. (2002). Choice is good, but relevance is excellent: autonomy-enhancing and suppressing teacher behaviours predicting students’ engagement in schoolwork. *Br. J. Educ. Psychol.* 72 261–278. 10.1348/000709902158883 12028612

[B5] Ben-Eliyahu A. Moore D. Dorph R. Schunn C. D. (2018). Investigating the multidimensionality of engagement: affective, behavioral, and cognitive engagement across science activities and contexts. *Contemp. Educ. Psychol.* 53 87–105. 10.1016/j.cedpsych.2018.01.002

[B6] Benita M. Roth G. Deci E. L. (2014). When are mastery goals more adaptive? It depends on experiences of autonomy support and autonomy. *J. Educ. Psychol.* 106 258–267. 10.1037/a0034007

[B7] Benita M. Shane N. Elgali O. Roth G. (2017). The important role of the context in which achievement goals are adopted: an experimental test. *Motiv. Emot.* 41 180–195. 10.1007/s11031-016-9600-8

[B8] Boekaerts M. de Koning E. Vedder P. (2006). Goal-directed behavior and contextual factors in the classroom: an innovative approach to the study of multiple goals. *Educ. Psychol.* 41 33–51. 10.1207/s15326985ep4101_5 33486653

[B9] Darnon C. Butera F. Harackiewicz J. M. (2007). Achievement goals in social interactions: learning with mastery vs. performance goals. *Motiv. Emot.* 31 61–70. 10.1007/s11031-006-9049-2

[B10] Deci E. L. Schwartz A. J. Sheinman L. Ryan R. M. (1981). An instrument to assess adults’ orientations toward control versus autonomy with children: reflections on intrinsic motivation and perceived competence. *J. Educ. Psychol.* 73:642 10.1037/0022-0663.73.5.642

[B11] Dompnier B. Darnon C. Butera F. (2009). Faking the desire to learn a clarification of the link between mastery goals and academic achievement. *Psychol. Sci.* 20 939–943. 10.1111/j.1467-9280.2009.02384.x 19538435

[B12] Dweck C. S. (1986). Motivational processes affecting learning. *Am. Psychol.* 41 1040–1048. 10.1037/0003-066X.41.10.1040

[B13] Elliot A. J. (1999). Approach and avoidance motivation and achievement goals. *Educ. Psychol.* 34 169–189. 10.1207/s15326985ep3403_3

[B14] Elliot A. J. (2005). “A conceptual history of the achievement goal construct,” in *Handbook of Competence and Motivation*, eds Elliot A. J. Dweck C. S. (New York, NY: Guilford Publications), 52–72.

[B15] Elliot A. J. McGregor H. A. (2001). A 2 x 2 achievement goal framework. *J. Pers. Soc. Psychol.* 80 501–519. 10.1037/0022-3514.80.3.501 11300582

[B16] Elliot A. J. Murayama K. (2008). On the measurement of achievement goals: critique, illustration, and application. *J. Educ. Psychol.* 100 613–628. 10.1037/0022-0663.100.3.613

[B17] Elliot A. J. Thrash T. M. (2001). Achievement goals and the hierarchical model of achievement motivation. *Educ. Psychol. Rev.* 13 139–156. 10.1023/A:1009057102306

[B18] Friedel J. M. Cortina K. S. Turner J. C. Midgley C. (2007). Achievement goals, efficacy beliefs and coping strategies in mathematics: the roles of perceived parent and teacher goal emphases. *Contemp. Educ. Psychol.* 32 434–458. 10.1016/j.cedpsych.2006.10.009

[B19] Gaudreau P. (2012). Goal self-concordance moderates the relationship between achievement goals and indicators of academic adjustment. *Learn. Individ. Differ.* 22 827–832. 10.1016/j.lindif.2012.06.006

[B20] Gonida E. N. Voulala K. Kiosseoglou G. (2009). Students’ achievement goal orientations and their behavioral and emotional engagement: co-examining the role of perceived school goal structures and parent goals during adolescence. *Learn. Individ. Differ.* 19 53–60. 10.1016/j.lindif.2008.04.002

[B21] Hagger M. S. Sultan S. Hardcastle S. J. Chatzisarantis N. L. D. (2015). Perceived autonomy support and autonomous motivation toward mathematics activities in educational and out-of-school contexts is related to mathematics homework behavior and attainment. *Contemp. Educ. Psychol.* 41 111–123. 10.1016/j.cedpsych.2014.12.002

[B22] Harackiewicz J. M. Barron K. E. Tauer J. M. Carter S. M. Elliot A. J. (2000). Short-term and long-term consequences of achievement goals: predicting interest and performance over time. *J. Educ. Psychol.* 92 316–330. 10.1037/0022-0663.92.2.316

[B23] Hu L. Bentler P. M. (1999). Cutoff criteria for fit indexes in covariance structure analysis: conventional criteria versus new alternatives. *Struct. Equ. Model. Multidiscipl. J.* 6 1–55. 10.1080/10705519909540118

[B24] Hughes D. J. (2018). “Psychometric validity,” in *The Wiley Handbook of Psychometric Testing: A Multidisciplinary Approach to Survey, Scale and Test Development*, eds Irwing P. Booth T. Hughes D. J. (Hoboken, NJ: Wiley), 751–779. 10.1002/9781118489772.ch24

[B25] Jang H. Kim E. J. Reeve J. (2012). Longitudinal test of self-determination theory’s motivation mediation model in a naturally occurring classroom context. *J. Educ. Psychol.* 104 1175–1188. 10.1037/a0028089

[B26] Jang H. Kim E. J. Reeve J. (2016). Why students become more engaged or more disengaged during the semester: a self-determination theory dual-process model. *Learn. Instruct.* 43 27–38. 10.1016/j.learninstruc.2016.01.002

[B27] Jang H. Reeve J. Deci E. L. (2010). Engaging students in learning activities: it is not autonomy support or structure but autonomy support and structure. *J. Educ. Psychol.* 102:588 10.1037/a0019682

[B28] Jang H. Reeve J. Ryan R. M. Kim A. (2009). Can self-determination theory explain what underlies the productive, satisfying learning experiences of collectivistically oriented Korean students? *J. Educ. Psychol.* 101 644–661. 10.1037/a0014241

[B29] Kaplan A. Middleton M. J. Urdan T. Midgley C. (2002). “Achievement goals and goal structures,” in *Goals, Goal Structures, and Patterns of Adaptive Learning*, ed. Midgley C. (New York, NY: Routledge), 21–53.

[B30] Kaplan H. (2018). Teachers’ autonomy support, autonomy suppression and conditional negative regard as predictors of optimal learning experience among high-achieving Bedouin students. *Soc. Psychol. Educ. Intern. J.* 21 223–255. 10.1007/s11218-017-9405-y

[B31] Krull J. L. Mackinnon D. P. (1999). Multilevel mediation modeling in group-based intervention studies. *Eval. Rev.* 23 418–444. 10.1177/0193841X9902300404 10558394

[B32] LeBreton J. M. Senter J. L. (2008). Answers to 20 questions about interrater reliability and interrater agreement. *Organ. Res. Methods* 11 815–852. 10.1177/1094428106296642

[B33] Lee W. Reeve J. (2012). Teachers’ estimates of their students’ motivation and engagement: being in synch with students. *Educ. Psychol.* 32 727–747. 10.1080/01443410.2012.732385

[B34] Levy I. Kaplan A. Patrick H. (2004). Early adolescents’ achievement goals, social status, and attitudes towards cooperation with peers. *Soc. Psychol. Educ.* 7 127–159. 10.1023/B:SPOE.0000018547.08294.b6

[B35] MacKinnon D. P. Lockwood C. M. Hoffman J. M. West S. G. Sheets V. (2002). A comparison of methods to test mediation and other intervening variable effects. *Psychol. Methods* 7:83. 10.1037/1082-989X.7.1.83 11928892 PMC2819363

[B36] Matos L. Lens W. Vansteenkiste M. Mouratidis A. (2017). Optimal motivation in Peruvian high schools: should learners pursue and teachers promote mastery goals, performance-approach goals or both? *Learn. Individ. Differ.* 55 87–96. 10.1016/j.lindif.2017.02.003

[B37] Matos L. Reeve J. Herrera D. Claux M. (2018). Students’ agentic engagement predicts longitudinal increases in perceived autonomy-supportive teaching: the squeaky wheel gets the grease. *J. Exper. Educ.* 86 592–609. 10.1080/00220973.2018.1448746

[B38] Michou A. Matos L. Gargurevich R. Gumus B. Herrera D. (2016). Building on the enriched hierarchical model of achievement motivation: autonomous and controlling reasons underlying mastery goals. *Psychol. Belgica* 56 269–287. 10.5334/pb.281 30479440 PMC5854211

[B39] Michou A. Vansteenkiste M. Mouratidis A. Lens W. (2014). Enriching the hierarchical model of achievement motivation: autonomous and controlling reasons underlying achievement goals. *Br. J. Educ. Psychol.* 84 650–666. 10.1111/bjep.12055 25251866

[B40] Midgley C. Anderman E. Hicks L. (1995). Differences between elementary and middle school teachers and students: a goal theory approach. *J. Early Adolesc.* 15 90–113. 10.1177/0272431695015001006

[B41] Midgley C. Kaplan A. Middleton M. (2001). Performance-approach goals: good for what, for whom, under what circumstances, and at what cost? *J. Educ. Psychol.* 93 77–86. 10.1037/0022-0663.93.1.77

[B42] Midgley C. Maehr M. L. Hruda L. Z. Anderman E. Anderman L. Freeman K. E. (2000). *Manual for the Patterns of Adaptive Learning Scales.* Ann Arbor: University of Michigan,

[B43] Mulvenna M. Adie J. W. Sage L. D. Wilson N. E. Howat D. (2020). Approach-achievement goals and motivational context on psycho-physiological functioning and performance among novice basketball players. *Psychol. Sport Exerc.* 51:101714 10.1016/j.psychsport.2020.101714

[B44] Muthén B. Asparouhov T. (2011). “Beyond multilevel regression modeling: multilevel analysis in a general latent variable framework,” in *Handbook of Advanced Multilevel Analysis*, eds Hox J. Roberts J. K. (New York, NY: Taylor & Francis), 15–40.

[B45] Muthén L. K. Muthén B. O. (2012). *User’s Guide*, 7th Edn, Los Angeles, CA: Muthén & Muthén.

[B46] Paulick I. Watermann R. Nückles M. (2013). Achievement goals and school achievement: the transition to different school tracks in secondary school. *Contemp. Educ. Psychol.* 38 75–86. 10.1016/j.cedpsych.2012.10.003

[B47] Pekrun R. Elliot A. J. Maier M. A. (2006). Achievement goals and discrete achievement emotions: a theoretical model and prospective test. *J. Educ. Psychol.* 98 583–597. 10.1037/0022-0663.98.3.583

[B48] Pelletier L. G. Séguin-Lévesque C. Legault L. (2002). Pressure from above and pressure from below as determinants of teachers’ motivation and teaching behaviors. *J. Educ. Psychol.* 94 186–196. 10.1037/0022-0663.94.1.186

[B49] Preacher K. J. Zyphur M. J. Zhang Z. (2010). A general multilevel SEM framework for assessing multilevel mediation. *Psychol. Methods* 15 209–233. 10.1037/a0020141 20822249

[B50] Raudenbush S. W. Bryk A. S. (2002). *Hierarchical Linear Models: Applications and Data Analysis Methods (Advanced Quantitative Techniques in the Social Sciences).* New York, NY: Sage.

[B51] Razer M. Mittelberg D. Motola M. Bar-Gosen N. (2015). Israeli high school teachers’ perceptions and attitudes towards a pedagogy of inclusion. *Intern. J. Inclusive Educ.* 19 944–964. 10.1080/13603116.2015.1019373

[B52] Reeve J. (2009). Why teachers adopt a controlling motivating style toward students and how they can become more autonomy supportive. *Educ. Psychol.* 44 159–175. 10.1080/00461520903028990

[B53] Reeve J. (2012). “A self-determination theory perspective on student engagement,” in *Handbook of Research on Student Engagement*, eds Christenson S. L. Reschly A. L. Wylie C. (Boston, MA: Springer), 149–172. 10.1007/978-1-4614-2018-7_7

[B54] Reeve J. (2016). “Autonomy-supportive teaching: what it is, how to do it,” in *Building Autonomous Learners: Perspectives from Research and Practice Using Self-Determination Theory*, eds Liu W. C. Wang J. C. K. Ryan R. M. (Singapore: Springer), 129–152. 10.1007/978-981-287-630-0_7

[B55] Reeve J. Jang H. (2006). What teachers say and do to support students’ autonomy during a learning activity. *J. Educ. Psychol.* 98 209–218. 10.1037/0022-0663.98.1.209

[B56] Reeve J. Jang H. Carrell D. Jeon S. Barch J. (2004). Enhancing students’ engagement by increasing teachers’ autonomy support. *Motiv. Emot.* 28 147–169. 10.1023/B:MOEM.0000032312.95499.6f

[B57] Ryan R. M. Connell J. P. (1989). Perceived locus of causality and internalization: examining reasons for acting in two domains. *J. Pers. Soc. Psychol.* 57 749–761. 10.1037/0022-3514.57.5.749 2810024

[B58] Ryan R. M. Deci E. L. (2017). *Self-Determination Theory: Basic Psychological Needs in Motivation, Development, and Wellness.* New York, NY: Guilford Press.

[B59] Ryan R. M. Deci E. L. (2000). Self-determination theory and the facilitation of intrinsic motivation, social development, and well-being. *Am. Psychol.* 55 68–78. 10.1037/0003-066X.55.1.68 11392867

[B60] Ryan R. M. Frederick C. (1997). On energy, personality, and health: subjective vitality as a dynamic reflection of well-being. *J. Per.* 65 529–565. 10.1111/j.1467-6494.1997.tb00326.x 9327588

[B61] Sarrazin P. G. Tessier D. P. Pelletier L. G. Trouilloud D. O. Chanal J. P. (2006). The effects of teachers’ expectations about students’ motivation on teachers’ autonomy-supportive and controlling behaviors. *Intern. J. Sport Exerc. Psychol.* 4 283–301. 10.1080/1612197X.2006.9671799

[B62] Satorra A. Bentler P. M. (2010). Ensuring positiveness of the scaled difference chi-square test statistic. *Psychometrika* 75 243–248. 20640194 10.1007/s11336-009-9135-y PMC2905175

[B63] Schuitema J. Peetsma T. van der Veen I. (2016). Longitudinal relations between perceived autonomy and social support from teachers and students’ self-regulated learning and achievement. *Learn. Individ. Differ.* 49 32–45. 10.1016/j.lindif.2016.05.006

[B64] Senko C. (2016). “Achievement goal theory: a story of early promises, eventual discords, and future possibilities,” in *Handbook of Motivation at School*, eds Wentzel K. R. Miele D. B. (New York, NY: Routledge), 87–107.

[B65] Senko C. Hulleman C. S. Harackiewicz J. M. (2011). Achievement goal theory at the crossroads: old controversies, current challenges, and new directions. *Educ. Psychol.* 46 26–47. 10.1080/00461520.2011.538646

[B66] Senko C. Tropiano K. L. (2016). Comparing three models of achievement goals: goal orientations, goal standards, and goal complexes. *J. Educ. Psychol.* 108 1178–1192. 10.1037/edu0000114

[B67] Shaffer J. A. DeGeest D. Li A. (2016). Tackling the problem of construct proliferation: a guide to assessing the discriminant validity of conceptually related constructs. *Organ. Res. Methods* 19 80–110. 10.1177/1094428115598239

[B68] Skinner E. A. Belmont M. J. (1993). Motivation in the classroom: reciprocal effects of teacher behavior and student engagement across the school year. *J. Educa. Psychol.* 85 571–581. 10.1037/0022-0663.85.4.571

[B69] Skinner E. A. Kindermann T. A. Furrer C. J. (2009). A motivational perspective on engagement and disaffection: conceptualization and assessment of children’s behavioral and emotional participation in academic activities in the classroom. *Educ. Psychol. Measur.* 69 493–525. 10.1177/0013164408323233

[B70] Soenens B. Sierens E. Vansteenkiste M. Dochy F. Goossens L. (2012). Psychologically controlling teaching: examining outcomes, antecedents, and mediators. *J. Educ. Psychol.* 104 108–120. 10.1037/a0025742

[B71] Sommet N. Elliot A. J. (2017). Achievement goals, reasons for goal pursuit, and achievement goal complexes as predictors of beneficial outcomes: is the influence of goals reducible to reasons? *J. Educ. Psychol.* 109 1141–1162. 10.1037/edu0000199

[B72] Spray C. M. John Wang C. K. Biddle S. J. H. Chatzisarantis N. L. D. (2006). Understanding motivation in sport: an experimental test of achievement goal and self-determination theories. *Eur. J. Sport Sci.* 6 43–51. 10.1080/17461390500422879

[B73] Tuominen-Soini H. Salmela-Aro K. Niemivirta M. (2012). Achievement goal orientations and academic well-being across the transition to upper secondary education. *Learn. Individ. Differ.* 22 290–305. 10.1016/j.lindif.2012.01.002

[B74] Vansteenkiste M. Lens W. Elliot A. J. Soenens B. Mouratidis A. (2014). Moving the achievement goal approach one step forward: toward a systematic examination of the autonomous and controlled reasons underlying achievement goals. *Educ. Psychol.* 49 153–174. 10.1080/00461520.2014.928598

[B75] Vansteenkiste M. Smeets S. Soenens B. Lens W. Matos L. Deci E. L. (2010). Autonomous and controlled regulation of performance-approach goals: their relations to perfectionism and educational outcomes. *Motiv. Emot.* 34 333–353. 10.1007/s11031-010-9188-3

[B76] Williams G. C. Deci E. L. (1996). Internalization of biopsychosocial values by medical students: a test of self-determination theory. *J. Per. Soc. Psychol.* 70:767. 10.1037/0022-3514.70.4.767 8636897

[B77] Wolters C. A. (2004). Advancing achievement goal theory: using goal structures and goal orientations to predict students’ motivation, cognition, and achievement. *J. Educ. Psychol.* 96 236–250. 10.1037/0022-0663.96.2.236

